# E-I balance emerges naturally from continuous Hebbian learning in autonomous neural networks

**DOI:** 10.1038/s41598-018-27099-5

**Published:** 2018-06-12

**Authors:** Philip Trapp, Rodrigo Echeveste, Claudius Gros

**Affiliations:** 1 Johann-Wolfgang-Goethe University Frankfurt, Frankfurt, 60323 Germany; 2 Computational and Biological Learning Lab, Dept. of Engineering, University of Cambridge, Cambridge, UK

## Abstract

Spontaneous brain activity is characterized in part by a balanced asynchronous chaotic state. Cortical recordings show that excitatory (E) and inhibitory (I) drivings in the E-I balanced state are substantially larger than the overall input. We show that such a state arises naturally in fully adapting networks which are deterministic, autonomously active and not subject to stochastic external or internal drivings. Temporary imbalances between excitatory and inhibitory inputs lead to large but short-lived activity bursts that stabilize irregular dynamics. We simulate autonomous networks of rate-encoding neurons for which all synaptic weights are plastic and subject to a Hebbian plasticity rule, the flux rule, that can be derived from the stationarity principle of statistical learning. Moreover, the average firing rate is regulated individually via a standard homeostatic adaption of the bias of each neuron’s input-output non-linear function. Additionally, networks with and without short-term plasticity are considered. E-I balance may arise only when the mean excitatory and inhibitory weights are themselves balanced, modulo the overall activity level. We show that synaptic weight balance, which has been considered hitherto as given, naturally arises in autonomous neural networks when the here considered self-limiting Hebbian synaptic plasticity rule is continuously active.

## Introduction

It is well established that a balance between excitation and inhibition, usually denoted as E-I balance, arises during spontaneous cortical activity, both *in vitro*^1–4^ and in the intact and spontaneously active cortex^4–7^. This balance, which refers to a relatively constant ratio between excitatory and inhibitory inputs to a neuron, has been theoretically predicted as way to explain how cortical networks are able to sustain stable though temporally irregular, and even chaotic, dynamics^8–10^. Since then, the ramifications of such a balanced state in terms of both dynamics and computation have been widely studied, showing how E-I balance results in critical-state dynamics of avalanches and oscillations^11^, with direct implications for the dynamic range^12^, storage of information^13^, and computational power^14^ of networks.

Recurrent neural networks can use E-I balance to generate asynchronous states even in the presence of strongly shared inputs^15^. Indeed, nearby cortical neurons with similar orientation tuning show low correlated variability, potentially simplifying the decoding of information by a population of such neurons^16^. Balanced networks have also been shown to work potentially in at least two different regimes, linking richness of the internal dynamics, connectivity strength, and functionality: a weak coupling state favoring information transmission, and a strongly coupled state, characterized by complex internal dynamics which could be employed for information processing^17^. Modulating the ratio between excitation and inhibition it is furthermore possible to selectively switch information gating and rerouting between different circuits on and off^18^.

The direct link between E-I balance and information transmission, together with observations of an atypical ratio of excitation/inhibition in neurobehavioral syndromes such as autism, has led to the hypothesis that an abnormal degree of E-I balance might be behind a series of psychiatric disorders^19^. Indeed, later causal experimental studies in mice have shown how further elevation of E-I balance, above typical physiological levels, produce a strong impairment of information processing and result in social deficits consistent with those of humans suffering from these conditions^20^.

It has been shown that networks of supralinear excitatory and inhibitory neurons, namely of neurons whose non-linearities are purely expansive (no saturation) and which would therefore tend to exhibit unstable behavior, can be stabilized choosing the right type of connectivity matrices, resulting in stabilized loosely balanced dynamics^21^. These networks, denoted stabilized supralinear networks (SSN), are able to capture a wide range of experimental findings of visual cortical neurons including contextual modulation and normalization, spatial properties of intracortical connections^22^, as well as stimulus dependence of neural variability^23^.

Different approaches have been taken in the past to construct balanced neural networks for numerical simulations. When van Vreeswijk and Sompolinsky introduced the balanced network model^8^ they constructed the connectivity matrix using sparse random connections, where the overall connection strength was forced to be inversely proportional to the square root of the number of connections. The conditions for stability of the (SSN) have also been studied analytically^21^, and the weights in these type of networks are typically selected so that the network is stable^22,23^. Balance has also been a topic of study in non-chaotic networks designed for generation of complex movement. Termed “stability-optimized circuits” (SOCs)^24^, in these networks balance is achieved by an optimizer modifying inhibitory connections, together with a mechanism able to prune or add new synapses.

These approaches did not however tackle the issue of how the brain could find those weight configurations. In particular, a key question is whether E-I balance in the brain is the result of genetically encoded synaptic strengths or, alternatively, whether ongoing internal synaptic adaption may lead to a dynamic configuration of balanced synaptic weights. In other words, whether the distribution of synaptic weights is a priori given or the result of a self-organizing process.

Several studies in recent years have proposed the use of inhibitory synaptic plasticity (ISP) to attain balance (see^25^, for a comprehensive review). Within those, an important step towards the understanding of how balance can be achieved in an unsupervised fashion in the brain has been the work of Vogels *et al*.^26^, who have shown in simulations how single neurons with constant (or controlled) E weights and plastic I weights, receiving an external stimulus can attain balance, and also restore it after the excitatory synaptic weights are modified. Moreover the authors also show how balance can be attained in random recurrent networks where only the connections from I cells to E cells are plastic via ISP.

Both excitatory and inhibitory connections, as well as the overall excitability of neurons are plastic and constantly evolve together with the neural activity in the brain, with a variety of plasticity mechanisms operating at different timescales^27–31^. The key question we address here is whether it would be possible for balance to be attained under these conditions in the brain, in a completely unsupervised way. We find here that this is indeed a plausible option. Namely, we show how simulated networks of non-linear excitatory and inhibitory neurons, evolving autonomously under local, online intrinsic and synaptic plasticity rules, generically achieve states which are balanced both with respect to the distribution of the synaptic weights and with respect to the inputs individual neurons receive. Hence we refer to this type of networks with continuously evolving synaptic and intrinsic parameters as Self-organized Plastic Balanced Network (SOPBN). No external optimization is here employed and the procedure is shown to be robust also to the addition of external noise.

## Methods

We consider autonomous Erdös-Rényi networks containing *N* neurons characterized by a linking probability *p*. The membrane potential *x*_*i*_ of the rate-encoding neurons obeys

1 $${\dot{x}}_{i}=\frac{1}{\tau }({x}_{i}^{(inp)}-{x}_{i}),\,{x}_{i}^{(inp)}=\sum _{j}\,{w}_{ij}{y}_{j},\,{y}_{i}=\frac{1}{1+{{\rm{e}}}^{{b}_{i}-{x}_{i}}},$$

where *y*_*i*_ is the firing rate, *b*_*i*_ the threshold and *w*_*ij*_ are the internal synaptic weights. There is no external input. In particular, no external source of noise is present in the main analysis of the system (we show in the Supplementary Material how these results are robust to the addition of a finite amount of external noise). The membrane time constant *τ* is set to 10 ms for inhibitory and respectively to 20 ms for excitatory neurons.

**Figure 1:**
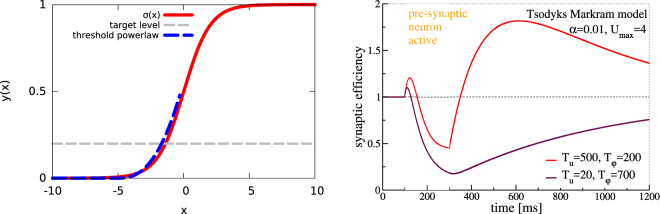
*Left*. In red the nonlinear transfer function relating membrane potentials and rates in the neural model (1). The typical activity rates enforced by the intrinsic plasticity rule (*y*_*t*_ = 0.2) result in the neuron operating at the foot of the non-linearity, where it is practically indistinguishable (*m*.*s*.*e*. = 0.027 for *x* ∈ [−5:0]) from a threshold-powerlaw with exponent *n* = 2.5 (in blue), typically considered a suitable model for experimental findings in cortical neurons^33–35^. *Right*. The effective synaptic strength multiplier *φ*(*t*)*u*(*t*) of the Tsodyks-Markram model (4). Here *β* = *α* = 0.01 and *U*_*max*_ = 4 was used. The red/violet curves correspond to the values as measured respectively for excitatory synapses in the medial prefrontal cortex of ferrets^43^ and for inhibitory layer 2–4 neurons of the somatosensory cortex layer of Wistar rats^44^. The presynaptic neuron is active for *t* ∈ [100, 300] (ms), and inactive otherwise.

The neural model we employ is described by a non-linear relation between membrane potentials and firing rates and has been used in previous work^32^ to derive the Hebbian plasticity rules we will later employ. This transformation is expansive for low firing rates and saturates for very high rates. While a saturation of this type is unavoidable for any realistic biological system, cortical neurons have always been observed to behave in the low firing rate regime, where this saturation is not visible, and the transfer function is typically described by a threshold-powerlaw $y\propto {\lfloor x\rfloor }^{n}$ with exponent *n* between 1 and 5^33–35^. We show however in Fig. 1 how, for low firing rates (encouraged by the intrinsic plasticity rule we employ) both functions are virtually indistinguishable.

### Adaption of the synaptic weights

The recurrent synaptic weights are continuously adapted using the multiplicative self-limiting Hebbian rule^32^

2 $${\dot{w}}_{ij}={\varepsilon }_{w}\,G({x}_{i})\,H({x}_{i}){y}_{j},\,G({x}_{i})={x}_{0}+{x}_{i}(1-2{y}_{i}),\,H({x}_{i})=2{y}_{i}-1+2{x}_{i}(1-{y}_{i}){y}_{i},$$

where the membrane potential *x*_*i*_ and the activity *y*_*i*_ of the postysynaptic neuron are related in this model via (1) by a deterministic sigmoidal transfer function. This allows us to write functions *G* and *H* as functions of *x*_*i*_ only, where *y*_*i*_ is then simply shorthand for *y*_*i*_(*x*_*i*_). This update rule may be derived from an information theoretical principle, the stationarity principle for statistical learning^36^, which states that the distribution function of the postsynaptic neural activity continuously evolves during the weight adaption process, becoming stationary only once learning is completed. Being autonomous the network considered here is however not confronted with an explicit learning task. Learning denotes in our context therefore the unsupervised process of weight adaption, which minimizes in our case the the Fisher information of the activity of the postsynaptic neuron^32^.

The limiting term *G*(*x*) in (2) changes sign when the postsynaptic activity *y*_*i*_ is either too large or too small in comparison with *x*_0_, reversing hence the Hebbian learning regulated in turn by *H*(*x*). This property of *G*(*x*) is useful for the learning rule as it prevents runaway synaptic growth, operating as an effective homeostatic synaptic plasticity mechanism, mounted on top of the Hebbian part of the rule^37^. Our adaption rule, which is also denoted flux rule^32^, is robust with respect to the actual value selected for the references scale *x*_0_ of the membrane potential, as we checked performing test runs with *x*_0_ = 1 and *x*_0_ = 8. For the simulations presented here we used *x*_0_ = 4.

We note that Hebbian learning rules like (2) are normally formulated not with respect to the bare presynaptic activities, but with respect to the deviation *δy*_*j*_ = *y*_*j*_ − 〈*y*_*j*_〉 of the presynaptic activity *y*_*j*_ with respect to its time-averaged mean〈*y*_*j*_〉. The adaption rule (2) performs in that case a principal component analysis for which the signal-to-noise ratio increases with increasing *x*_0_^32^, being otherwise sensible to input directions *y*_*j*_ characterized by a negative excess kurtosis.

For the study presented further below we use the same adaption rule for all synapses, namely (2), whose self-limiting behavior stabilizes firing rates, rather than trying to reproduce a particular instance of the wide variety of experimentally observed phenomenological spike time dependent synaptic plasticity (STDP) rules for inhibitory connections^25^. This route would involve therefore the introduction of not well-constrained parameters, transcending in addition the central aims of our investigation. We are interested here to investigate if ongoing Hebbian plasticity and balanced asynchronous dynamics are compatible.

The threshold *b*_*i*_ = *b*_*i*_(*t*) entering the transfer function in (1) sets, as usual, the average firing rates. Here we use

3 $${\dot{b}}_{i}={\varepsilon }_{b}({y}_{i}-{y}_{t})$$

for the adaption rule for the threshold, which reduces, for *y* ≈ *y*_*t*_ = 0.2, to the somewhat extended expressions one may derive from homeostatic principles for neural activity^38–40^. For the adaption rates we used 1/*ε*_*b*_ = 10 and 1/*ε*_*w*_ = 100 (in seconds).

### Synaptic pruning

Dale’s law states that neurons are either excitatory or inhibitory, namely that *w*_*lj*_*w*_*kj*_ ≥ 0 for all *l* and *k*. For a Hebbian plasticity rule like (2) to respect Dale’s law one needs to prune a synaptic connection whenever the respective *w*_*ij*_ changes sign. We do this every 1000 ms of mathematical simulation time, reinserting the pruned link with a weight corresponding to 10% of the correspondingly average excitatory or inhibitory links. Performing test runs where the pruned links were reinserted with a strength of 1% of the average mean yielded nearly identical results. For the reinsertion process the postsynaptic neuron *i* is connected to a random and previously unconnected presynaptic neuron *m*, with the sign of the new link *w*_*im*_ respecting Dale’s law. There are two possible versions.

#### Annealed pruning

Links may change sign when the new presynaptic neuron *m* is selected freely. The overall number of excitatory and inhibitory links may then drift over the course of the simulation, with only the total connectivity remaining constant.

#### Frozen pruning

Links do not change in character when the new presynaptic neuron *m* is selected only among those neurons which are of the same type as *j*. Frozen pruning would correspond from a biological perspective to a separate reshuffling of Gaba and Glutamate receptors.

For the results presented here we considered frozen pruning.

### Short-term synaptic plasticity

We also included short-term plasticity (STSP), a mostly presynaptically induced modulation of the synaptic efficacy lasting hundreds of milliseconds to seconds^41^. STSP may lead both to synaptic potentiation and depression, resulting respectively from an influx of Ca^2+^ ions into the presynaptic bulb and from a depletion of the available reservoir of neurotransmitters. These effects are captured within the Tsodyks-Markram model^42^ by two variables, *u*(*t*) and *φ*(*t*), encoding respectively the presynaptic Ca^2+^-concentration and the number of vesicles with neurotransmitters. The transient plasticity rules

4 $${\dot{u}}_{j}=\frac{1-{u}_{j}}{{T}_{u}}+\alpha ({U}_{max}-{u}_{j}){y}_{j},\,{\dot{\phi }}_{j}=\frac{1-{\phi }_{j}}{{T}_{\phi }}-\beta {\phi }_{j}{u}_{j}{y}_{j},\,{\mathop{w}\limits^{ \sim }}_{ij}={w}_{ij}{\phi }_{j}(t){u}_{j}(t)$$

then describe the time evolution of the effective synaptic weight ${\tilde{w}}_{ij}$ which is proportional to the bare synaptic weight *w*_*ij*_, to the number of available vesicles *φ*_*j*_ and to the vesicle’s release probability *u*_*j*_. In simulations where STSP is present, ${\tilde{w}}_{ij}$ replaces *w*_*ij*_ in (1). STSP is transient in the sense that both *u*_*j*_ and *φ*_*j*_ relax to unity in the absence of presynaptic activity *y*_*j*_ → 0. Typical time evolution curves for the synaptic efficiency multiplier *φ*_*j*_(*t*)*u*_*j*_(*t*) are presented in Fig. 1.

With the introduction of STSP and making an explicit distinction between E and I inputs, the driving current ${x}_{i}^{(inp)}$ defined in (1) is then generalized to

5 $${x}_{i}^{(inp)}={x}_{i}^{(exc)}+{x}_{i}^{(inh)},\,{x}_{i}^{(exc)}=\sum _{j\in \{exc\}}\,{w}_{ij}{\phi }_{j}{u}_{j}{y}_{j},\,{x}_{i}^{(inh)}=\sum _{j\in \{inh\}}\,{w}_{ij}{\phi }_{j}{u}_{j}{y}_{j},$$

where {*exc*} and {*inh*} denote respectively the set of excitatory and inhibitory neurons. One can define analogously with

6 $${\bar{w}}^{(exc)}=\frac{{\sum }_{i,j\in \{exc\}}{w}_{ij}{\phi }_{j}{u}_{j}}{{\sum }_{i,j\in \{exc\}}},\,{\bar{w}}^{(inh)}=\frac{{\sum }_{i,j\in \{inh\}}{w}_{ij}{\phi }_{j}{u}_{j}}{{\sum }_{i,j\in \{inh\}}}$$

the average excitatory and inhibitory effective synaptic weights.

We note that the original Tsodyks-Markram model^42^ describes STSP for the case of spiking neurons and that one can derive (4) by assuming *α* = *β* = 0.01 and that a maximal neural activity of *y*_*j*_ → 1 corresponds to a firing rate of 40 *Hz*. Typical values for the time scales entering (4) are *T*_*u*_ = 500 ms and *T*_*φ*_ = 200 ms for excitatory synapses in the medial prefrontal cortex of ferrets^43^ and *T*_*u*_ = 20 ms and *T*_*φ*_ = 700 ms for inhibitory layer 2–4 neurons of the somatosensory cortex of Wistar rats^44^. It has been pointed out, that these time scales are also relevant for behavioral control tasks^45^.

For our simulations we used *U*_*max*_ = 4, *α* = *β* = 0.01, *T*_*u*_ = 500 ms and *T*_*φ*_ = 200 ms for all synapses. We did also run control runs involving 500/200 and 20/700 *T*_*u*_/*T*_*φ*_ pairs respectively for excitatory and inhibitory synapses, which led however only to minor quantitative changes.

## Results

We are interested in investigating under which conditions an autonomous neural network, whose dynamics is described by (1), (2), (3) and (4), evolves towards a stable, irregular and balanced state (SOPBN). The results here presented correspond to networks of both excitatory and inhibitory neurons, where 80% of neurons are excitatory and 20% are inhibitory, and whose connections respect Dale’s principle, even when plasticity mechanisms are at play. We have taken membrane time constants of 20 and 10 ms for excitatory and inhibitory cells, respectively. As checks, we have also repeated the simulations with networks consisting of 50% excitatory and 50% inhibitory neurons and with equal membrane time constants, observing no qualitative differences. Unless otherwise stated, we will present results with a total number of neurons *N* = 400, a fixed 80% fraction of excitatory cells, a link probability *p* = 0.2 and a target average activity of *y*_*t*_ = 0.2. The initial synaptic weights are drawn from Gaussians with means 7.5 (−30.0) and standard deviations 0.375 (1.5) for excitatory and inhibitory synapses, respectively. Our simulations were performed in all cases with a C++ code running on a standard desktop computer.

### Rate encoding neurons with asynchronous activity spikes

**Figure 2:**
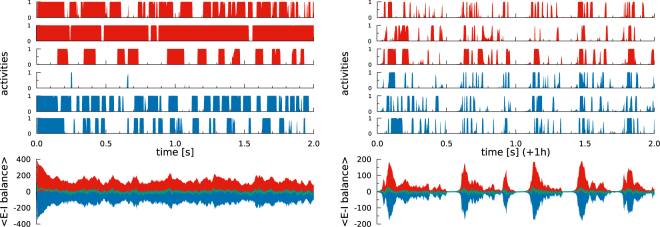
Selected activities and the average input current for an autonomous SOPBN containing 320 (80) excitatory (inhibitory) rate-encoding neurons, as defined by (1), (2), (3) and (4). The target activity is *y*_*t*_ = 0.2. The time interval is two seconds and the system is shown for the first two seconds after initialization *(left)*, and for two seconds after a previous evolution of 3600 seconds of simulated biological time *(right)*. Shown are the activities of three random excitatory (red) and inhibitory (blue) neurons, together with the averaged E-I balance. The E-I balance is given here in terms of the network-average of excitatory $\langle {x}_{i}^{(exc)}\rangle$ and inhibitory $\langle {x}_{i}^{(inh)}\rangle$ inputs (red and blue curves), as defined by (5). The sum (green) is substantially smaller in magnitude for both time intervals. While the neural activities show a large spectrum of activities reaching from nearly completely silent to almost constantly firing neurons at the beginning, the averaged activity after 1 h shows irregular bursts which are characterized by asynchronous neural activities. Note that the large fluctuations in the inputs of the rate-encoding neurons making up the network induce ‘spike like’ activities.

We find that the SOPBN tends to evolve to an irregularly bursting state characterized by time scales of the order of 100–200 ms. The data presented in Fig. 2 illustrates typical two second intervals of activity, as obtained directly at initialization and after one hour of mathematical simulation time. It shows the following:

- The system state is very different at the beginning and after one hour: While some neurons are constantly quiet or active directly after initialization, the network exhibits pervading bursts after evolving for one hour.
- The mean excitatory $\langle {x}_{i}^{(exc)}\rangle$ and inhibitory $\langle {x}_{i}^{(inh)}\rangle$ inputs a neuron receives are both large in magnitude. The substantially smaller value for the overall mean input expresses E-I balance. This E-I balance is present for arbitrary timeframes within the systems evolution. Averaged over time we have
  $$\begin{array}{c}{\langle {x}_{i}^{(exc)}\rangle }_{t=0{\rm{s}}}\approx 144.8,\,{\langle {x}_{i}^{(inh)}\rangle }_{t=0{\rm{s}}}\approx -\,147.3,\,{\langle {x}_{i}^{(exc)}\rangle }_{t=0{\rm{s}}}+{\langle {x}_{i}^{(inh)}\rangle }_{t=0{\rm{s}}}\approx -\,2.5,\\ {\langle {x}_{i}^{(exc)}\rangle }_{t=1{\rm{h}}}\approx 41.9,\,{\langle {x}_{i}^{(inh)}\rangle }_{t=1{\rm{h}}}\approx -\,44.1,\,{\langle {x}_{i}^{(exc)}\rangle }_{t=1{\rm{h}}}+{\langle {x}_{i}^{(inh)}\rangle }_{t=1{\rm{h}}}\approx -\,2.2,\end{array}$$
       for the system at different times where the brackets denote now averages over the network and over time.
- Deviations from the average E-I balance lead to large swings in the membrane potentials and hence to sharp activity spikes. A remarkable feature for rate-encoding neurons evolving with (1) continuously in time.
- Bursts in the late network state involve the entire network. All excitatory and inhibitory neurons are active one or more times during a burst, as we have checked. The activities of the individual neurons are however asynchronous.

We also examined the E-I balance ${x}_{i}^{(exc)}+{x}_{i}^{(inh)}$ for individual neurons, obtaining results very close to the network averages shown in Fig. 2. A detailed analysis of the corresponding cross correlations is presented further below.

In^21^ the authors compare the degree of cancellation (or tightness of the balance) between the van Vreeswijk and Sompolinsky balanced networks, and the SSN, showing that while the first kind requires a very high degree of cancellation, the SSN can operate in a regime of loose balance. These networks have however constant synaptic weights and intrinsic parameters. We observe in SOPBNs, where several parameters are plastic, that while most of the time the network follows a high degree of balance (with correlations close to unity as shown in Fig. 7), this tightness is transiently broken to allow for bursts of activity.

**Figure 3:**
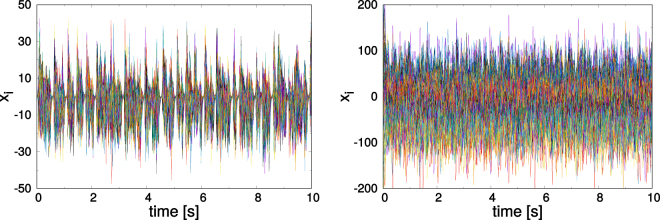
The superimposed 10 sec traces of the membrane potentials of a network of *N* = 400 neurons and a relative fraction of excitatory to inhibitory neurons of 80:20. *Left*: After a simulation of 3600 sec for the same SOPBN considered in Figs. 2 and 4. *Right*: For a network with only short-term synaptic plasticity, namely with *ε*_*b*_ = 0 = *ε*_*w*_. Note that the synaptic weights are in this case as drawn from the initial distribution, which is balanced with means 7.5 (−30.0) and widths 0.375 (1.5) for excitatory and respectively for inhibitory synapses. Additionally turning off short-term synaptic plasticity changes the irregular state only quantitatively.

Autonomous networks with balanced and increasingly large, but otherwise random synaptic weight distributions, are known to produce a chaotic state in the thermodynamic limit^9^. Testing this prediction we considered the non-adapting case with *ε*_*b*_ = *ε*_*w*_ = 0. By additionally switching off short-term synaptic plasticity, we find that a *N* = 400 network leads, depending on the initial weight distribution, either to fixpoints, limit-cycles, or to states of highly irregular activity. We however did not try to determine the relative incidence rates of theses three states. The two types of irregular spiking states, which are illustrated in Fig. 3, as resulting from adapting and from non-adapting dynamics, differ with respect to activity bursts (which are observed also in Fig. 2), which are conspicuously absent in our non-adapting networks.

As a note, these irregular spiking states show signs of corresponding to a transient chaotic state (see subsection *Analysis of the irregular activity*, in the Supplementary Material).

### Evolution of balanced synaptic weights

**Figure 4:**
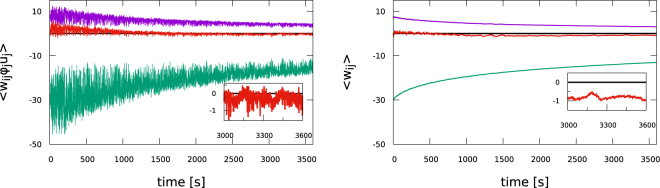
Time evolution of the average effective excitatory (violet) and inhibitory (green) synaptic weights ${\overline{w}}^{(exc)}$ and ${\overline{w}}^{(inh)}$, as defined by (6). The network contains 320 and 80 excitatory and inhibitory neurons. Also shown is the average balanced weight (red, enlarged in the insets), given by $4{\overline{w}}^{(exc)}+{\overline{w}}^{(inh)}$. *Left*: With short-term plasticity. *Right*: Without short-term plasticity, namely for *φ*_*j*_ ≡ 1 and *u*_*j*_ ≡ 1.

We present in Fig. 4 the evolution of the network averages (6) of the synaptic weights. We find that the Hebbian plasticity rule (2) renormalizes the synaptic weights while approximately retaining the balance

7 $${f}_{exc}{\bar{w}}^{(exc)}\langle {y}_{i}^{(exc)}\rangle \approx {f}_{inh}{\bar{w}}^{(inh)}\langle {y}_{i}^{(inh)}\rangle ,\,4{\bar{w}}^{(exc)}\approx -\,{\bar{w}}^{(inh)}$$

between the mean excitatory ${\overline{w}}^{(exc)}$ and the mean inhibitory ${\overline{w}}^{(inh)}$ weights, where we have denoted with *f*_*exc*_/*f*_*inh*_ and $\langle {y}_{i}^{(exc)}\rangle /\langle {y}_{i}^{(inh)}\rangle$ the fractions and the mean activities of excitatory and inhibitory neurons. For the present study we have $\langle {y}_{i}^{(exc)}\rangle =\langle {y}_{i}^{(inh)}\rangle ={y}_{t}$. The second relation in (7) refers to 80/20 networks, which contain four times as many excitatory as inhibitory neurons.

- The balance presented in Fig. 4 is not perfect, with the inhibitory weights being slightly dominating on the long run.
- We also considered networks for which the initial weight distribution was strongly not balanced, finding that the adaption rule (2) leads to balanced mean synaptic weights. We will discuss the self organization of E-I balance in more detail further below for the case of 50/50 networks.

**Figure 5:**
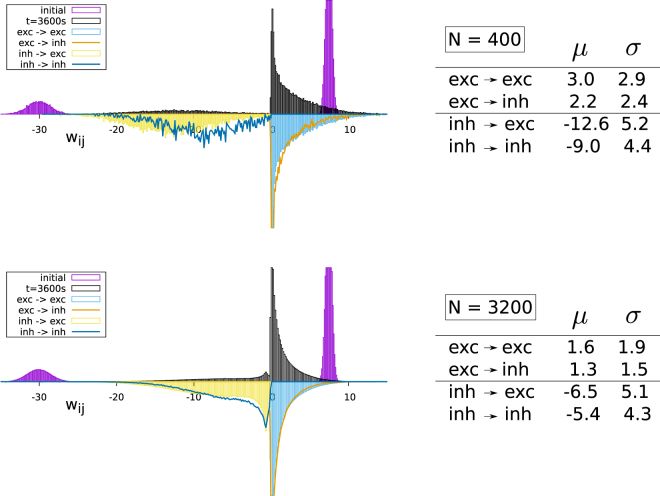
*Top*: The distribution of the synaptic weights *w*_*ij*_ for *N* = 400 site networks with a link probability of *p* = 0.2 and 80% excitatory and 20% inhibitory neurons. *Bottom*: The same for *N* = 3200 neurons. Shown are in the right panels the histograms of the initial distribution (violet, top part truncated) and the distribution as obtained after a mathematical simulation time of 3600 seconds. Black: the overall distribution of synaptic weights and (reflected with regard to the x-axis) the individually normalized partial distributions (excitatory/inhibitory) → (excitatory/inhibitory) neurons. The respective means *μ* and standard deviations *σ* of the partial distributions are given in the panels on the right.

In Fig. 5 the full distribution of synaptic weights is presented, with the results obtained from a 3600 sec simulation contrasted to the initial weight distribution. It is evident that the redistribution of synaptic weights is substantial, reaching far beyond a simple overall rescaling of the mean, as presented in Fig. 4. The excitatory weights, and to a certain extent also the inhibitory weights, tend to pile up at the pruning threshold, which has been set to zero. Trying exponential and log-normal fits we found that the excitatory weight distribution follows fairly well a log-normal distribution.

### System size and simulation time effects

The comparison between networks with *N* = 400 and *N* = 3200 presented in Fig. 5 shows that the overall functional form of the weight distribution changes qualitatively for the inhibitory weights, but not for the excitatory weights. The small additional peak visible for *N* = 3200 for the inhibitory links corresponds to the synaptic weights of the links reinserted after pruning.

The mean weights, which are also presented in Fig. 5, scale down with increasing systems size. For the data presented in Fig. 5 the connection probability is *p* = 0.2 for both *N* = 400 and *N* = 3200. It is then an interesting question which kind of scaling autonomous Hebbian learning would produce. Our attempts to determine how the synaptic weights scale with respect to the mean number of afferent synapse *Z* = *pN* were however not successful. For the data presented in Fig. 5 we note that the ratio of the mean synaptic weights is about a factor two for *N* = 400 and *N* = 3200, with the corresponding ratio of *Z* being 1/8.

Comparing weight distributions for a fixed simulation time is not meaningful for systems, as our SOPBN, that do not stop evolving. Average weights continue to drop even for long-term simulations, as evident in part in Fig. 4. We find that the system switches to a new state (characterized either by limit cycles, fixpoints or by very long quiet periods) after extended transients, which are at least of the order of several hours. The irregular state observed, as in Fig. 3, corresponds therefore to a transient state. The transients last however orders of magnitude longer than the time scales relevant for information processing in biological networks, which range typically from milliseconds to seconds.

### Self-organized balanced synaptic weights

The results presented hitherto in Figs 2, 3, 4 and 5 have been for 80/20 systems where the initial synaptic weights had been drawn from balanced distributions. Going one step further we now examine whether the Hebbian plasticity rule (2) is able to transform a non-balanced weight distribution into a balanced distribution.

**Figure 6:**
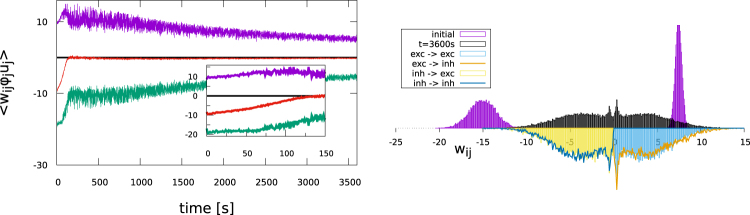
*Left*: The evolution of the effective synaptic weights, as for Fig. 4, but for 200 excitatory and 200 inhibitory neurons. The membrane integration time in (1) is set to *τ* = 20 ms for both excitatory and inhibitory neurons. Synaptic weight balance (7), as expressed by ${\overline{w}}^{(exc)}+{\overline{w}}^{(inh)}$ (red curve), is achieved on the time scale 1/*ε*_*w*_ = 100 s of Hebbian learning (see inset). Note that the initial synaptic weight balance has been selected to be off by a factor of two. *Right*: The synaptic weight distributions, as for Fig. 5, obtained after one hour of mathematical simulation time. The two small peaks are located at the value for the weight reinserted after pruning. The final distributions are symmetric, apart from some stochastic fluctuations, with standard deviations of 2.7 and means of ±4.1 for excitatory and inhibitory neurons. Note that the initial weight distribution (violet) is highly unbalanced.

We present in Fig. 6 the evolution of the synaptic weights for a 50/50 system, for which the initial synaptic weights had been drawn from Gaussians with means 7.5 (−15.0) and standard deviations 0.375 (1.5) for excitatory and inhibitory synapses, respectively. One notices that the autonomous Hebbian learning rule (2) balances the initially unbalanced synaptic weight distribution as fast as possible, that is, on the timescale 1/*ε*_*w*_ = 100 s. Equivalent results were obtained for initially unbalanced 80/20 systems.

**Figure 7:**
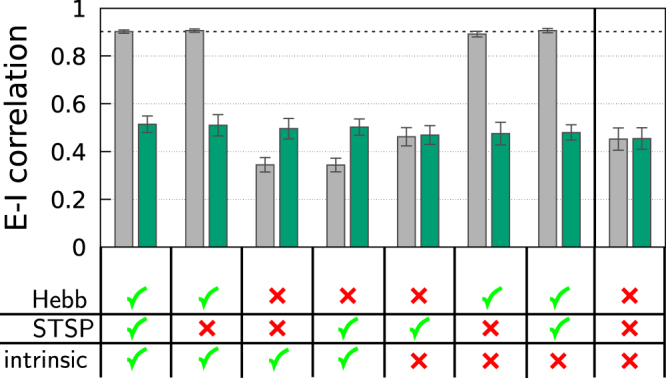
The E-I cross-correlation between excitatory and inhibitory inputs for a 50/50 system with *N* = 400 neurons. Shown is |*ρ*^±^| = −*ρ*^±^, as defined in (10), which was measured either after 1 hour (gray bars), or right at the start (green bars). For the time average a period of 10 sec has been used in both cases. The error bars have been evaluated with respect to 100 initial weight configurations drawn each time from Gaussians with means 7.5 (−15.0) and standard deviations 0.375 (1.5) for excitatory and inhibitory synapses, respectively. The initial synaptic weight configuration is therefore not balanced (as for Fig. 6). Shown are the results for distinct scenarios with Hebbian plasticity (Hebb), short-term synaptic plasticity (STSP) and intrinsic plasticity (intrinsic) being either turned on (green checkmark) or off (red cross).

The distribution of synaptic weights self-organizes, as evident from the data presented in Fig. 6, becoming fully symmetric within one hour of Hebbian adaption. The same is found for initially non-balanced 80/20 networks (not shown), for which the final synaptic weight is also balanced, albeit non-symmetric.

### Would any Hebbian learning rule lead to balanced synaptic weights?

A range of distinct synaptic plasticity rules are Hebbian in the sense that they perform a principal component analysis (PCA) whenever a direction in the space of input activities presents a larger variance with respect to all other input directions^32^. Examples are the flux rule (2), which may be derived from the stationarity principle for statistical learning^36^, and Oja’s rule^46^,

8 $${\dot{w}}_{ij}={\varepsilon }_{{\rm{o}}{\rm{j}}{\rm{a}}}{y}_{i}({y}_{j}{\phi }_{j}{u}_{j}-\alpha {y}_{i}{w}_{ij}),\,\alpha =\mathrm{0.1.}$$

In order to work with average synaptic weight changes $\langle {\dot{w}}_{ij}\rangle$ of comparable magnitude, one needs to rescale the adaption rate *ε*_oja_ with respect to *ε*_*w*_, which enters the flux rule (2). We use *ε*_oja_ = 10*ε*_*w*_.

**Figure 8:**
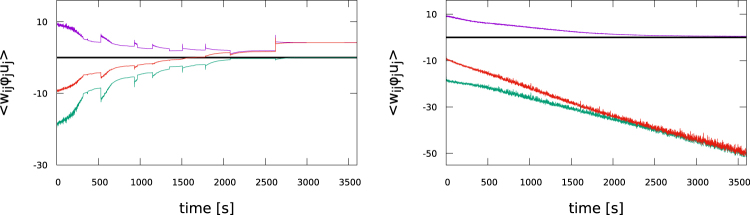
Time evolution of the average effective excitatory (violet) and inhibitory (green) synaptic weights ${\overline{w}}^{(exc)}$ and ${\overline{w}}^{(inh)}$, as defined by (6). The network contains 200 and 200 excitatory and inhibitory neurons. Also shown is the average balanced weight (red), given by ${\overline{w}}^{(exc)}+{\overline{w}}^{(inh)}$. *Left*: Using Oja’s rule (8). *Right*: Using the flux rule (2), as for Fig. 6, but this time with the limiting factor *G*(*x*) = *x*_0_ + *x*(1 − 2*y*) replaced by a constant, *G* → 10. Both approaches fail to produce a balanced synaptic weight distribution.

In Fig. 8 the time evolution of the average excitatory and inhibitory synaptic weights, as produced by Oja’s rule (8), are presented. Oja’s rule leads to a complete rescaling of the inhibitory weights and hence to a maximally unbalanced synaptic weight distribution, which is furthermore characterized by intermittent periods of abrupt changes.

Synaptic weight growth is limited by both Oja’s and by the flux rule, namely as a consequence of the additive damping factor for the case of Oja’s rule (8) and as the result of the multiplicative limiting factor *G*(*x*) = *x*_0_ + *x*(1 − 2*y*) for the case of the flux rule (2). For comparison we performed simulations where we replaced *G*(*x*) in (2) by a constant. We find in this case that the excitatory weights are rescaled to zero. The synaptic weight distribution is therefore also maximally unbalanced. The runaway growth of the inhibitory synaptic weights showing up in Fig. 8, which is due to the removal of the limiting factor *G*(*x*) in (2), is accompanied by a respective evolution of the threshold, via (3), such that the average activity remains close to *y*_*t*_ = 0.2.

The flux rule (2) is manifestly only a function of the membrane potential *x*_*i*_ and of the effective presynaptic activity *φ*_*j*_*u*_*j*_*y*_*j*_, which is in turn positive. The overall functional form follows closely that of a cubic polynomial^36^,

9 $${\dot{w}}_{ij}\approx -\,{\varepsilon }_{w}({x}_{i}-b/2)\,({x}_{i}-{x}^{-})\,({x}_{i}-{x}^{+})\,{\phi }_{j}{u}_{j}{y}_{j},\,{x}^{\pm }\approx -\,\frac{{b}_{i}}{2}\pm {x}_{0},$$

where the *x*^±^ denote the roots of *G*(*x*) = *x*_0_ + *x*(1 − 2*y*). Stationarity is achieved when the time average of (9) vanishes, that is when the average membrane potential 〈*x*_*i*_〉 is on the order of the size of the roots *x*^±^ and *b*/2 of *G*(*x*)*H*(*x*).

- The threshold *b*, which is determined via the sigmoidal (1) by the target activity *y*_*t*_, is of order unity whenever this is the case for the average membrane potential 〈*x*_*i*_〉.
- It is viceversa true, that the average membrane potential 〈*x*_*i*_〉 will be of order unity, as long as this is the case for the roots *x*^±^ and *b*/2 of *G*(*x*)*H*(*x*).

These two conditions are mutually compatible. It is from this point not surprising that the flux rule leads on average to small membrane potentials, as evident in Fig. 3, and consequently also to approximately balanced synaptic weight distributions. We note in contrast that Oja’s rule (8) is explicitly dependent in addition on the weight *w*_*ij*_ of the adapting synapse.

We conclude that not every Hebbian learning rule will produce balanced irregular dynamics. While we have pointed out here at some differences between the Flux rule and Oja’s rule, which may hint at the conditions for a rule to achieve this state, further work is necessary to determine which families of rules can and cannot perform this task.

### E-I balance in terms of E-I correlations

To now quantify the degree of balance between excitation and inhibition, we compute for a given neuron the cross-correlation ${C}_{i}^{\pm }$ between the total excitatory incoming synaptic current ${x}_{i}^{(exc)}$, as defined by (5), and the total inhibitory synaptic current ${x}_{i}^{(inh)}$, averaged first with respect to time and then across all neurons of the network:

10 $${C}_{i}^{\pm }=\frac{{\langle ({x}_{i}^{(exc)}-{\langle {x}_{i}^{(exc)}\rangle }_{t})({x}_{i}^{(inh)}-{\langle {x}_{i}^{(inh)}\rangle }_{t})\rangle }_{t}}{{\sigma }_{i}^{(exc)}{\sigma }_{i}^{(inh)}},\,{\rho }^{\pm }={\langle {C}_{i}^{\pm }\rangle }_{i}.$$

Here we have denoted with ${\sigma }_{i}^{(exc)}$ and ${\sigma }_{i}^{(inh)}$ the standard deviations of ${x}_{i}^{(exc)}$ and respectively of ${x}_{i}^{(inh)}$.

In Fig. 7 we present the cross correlation |*ρ*^±^| for the 50/50 system discussed in Fig. 6, for which the initial weight configurations are not balanced. Note that the time scale for Hebbian learning is 1/*ε*_*w*_ = 100 sec, which is a order of magnitude larger than the interval of 10 sec used for evaluating *ρ*^±^ via (10). Analogous investigations for an 80/20 system can be found in the Supplementary Material in Fig. S3.

The cross correlation characterizing the E-I balance of the initial state is only marginally dependent on whether short-term and/or intrinsic plasticity are active. Its surprisingly large overall value, about (45–50)%, reflects the presence of substantial inter-neuronal activity correlations, which we did not investigate further. Comparing with the data presented in Fig. 6 one notices that *ρ*^±^ is a somewhat less sensible yardstick for E-I balance than the bare synaptic weight balance, which renormalizes to small values in a balanced state. The data shown in Fig. 7 confirms otherwise that the Hebbian plasticity rule (2) leads to a highly balanced state.

We have so far considered here networks without any external noise, which would not be the case in the brain. A state characterized by irregular neural activity is generically expected to be robust against moderate noise levels. Performing simulations with additive input noise, characterized by zero means and a standard deviation of (5–10)%, with respect to the mean of the bare input, we found this expectation to hold. The cross correlation *ρ*^±^ barely changes as long as the level of noise present remains moderate. The situation changes gradually with increasing noise strength, with E-I balance breaking down when the noise level reaches about 50% of the bare input strength (cf. Fig. S1 in the Supplementary Material).

## Discussion

We have examined here the question of whether it would be plausible for a neural network in which both intrinsic and synaptic (E as well as I connections) parameters are continuously evolving to achieve balance both in terms of weights and activities, in a fully unsupervised way, finding that this is indeed possible. The resulting balanced network (which we have denoted here SOPBN) arises in a self-organized fashion, in analogy to the critical state characterizing possibly certain aspects of cortical dynamics^47^. We studied for this purpose the influence of continuously ongoing Hebbian plasticity within autonomous networks of rate-encoding neurons, finding that the synaptic plasticity rule that follows from the stationarity principle of statistical learning, the flux rule, does indeed induce a balanced synaptic weight distribution, even when the initial distribution is strongly unbalanced.

### E-I balance induced by Hebbian learning

Comparing the flux rule with and without the self-limiting term and Oja’s rule, we have found that Hebbian learning leads to a balanced distribution of synaptic weights, and hence also to a balanced state, whenever the learning rule favors small average membrane potentials. It is not necessary, for this to happen, that the learning rule constrains the overall input to strictly vanish on average, it suffices that the time averaged input remains of the order of the neural parameters, such as the inverse slope of the transfer function in (1). We found that the flux rule, as defined by (2) and (9), fulfills this requirement. An example of a Hebbian rule not leading to a balanced weight distribution is on the other side given by Oja’s rule (8).

### Rate encoding neurons showing spike-like neural activity

An E-I balanced state is characterized in addition to the small average membrane potential by the near cancellation of two large drivings in the form of large excitatory and inhibitory inputs. Such a state is highly sensible to small imbalances resulting either from additional external signals or from internal fluctuations. We find these imbalances to be strong enough in SOPBNs to induce short spike-like bursts in the neural activity, as observed e.g. in Fig. 2. This is quite remarkable, as one could have expected that the rate-encoding neurons used for the present study would be more likely to lead to slowly and hence to smoothly varying dynamical states.

### Asynchronous neural activity

The near cancellation of large excitatory and inhibitory drivings stabilizes asynchronous neural activity, as illustrated in Fig. 3 in terms of the membrane potential. Using the 0–1 test for chaos^48^ we found the asynchronous state in SOPBNs to be at least strongly irregular (cf. Fig. S2 in the Supplementary Material). As indicators for chaos one may have analyzed the time intervals between activity spikes^49^ or the Lyapunov exponents of the system. The observation that the synaptic weight distribution changes continuously, as demonstrated in Fig. 6, over time scales of hours, proves in any case that the neural activity is irregular on extended times scales. The limit of infinitely long times is not the focus of this study, as real neural systems are not expected to function for prolonged periods in the absence of stimuli.

### Absence of a stationary autonomous state

We find, as shown in Fig. 4, that the size of the mean synaptic weights decays slowly but continuously. Experimenting with different ensembles of initial weight statistics we found no instance where Hebbian learning retaining E-I balance would lead to a systematic increase in magnitude of the overall mean synaptic weights. We note, however, that this observation holds only for the here considered case of isolated networks, hence without an additional external driving. An adaption rate *ε*_*w*_ that would fade out slowing, being only initially large, would also preempt the long term decay of average synaptic weights.

### Theory vs. experiment

The dynamic balance of excitation and inhibition is observed experimentally within a range of distinct settings^1,5^. Multielectrode recordings in human and monkey neocortex suggests that E-I balance is caused in essence by local recurrent activity^50^, and not by external inputs, with irregular bursting activity showing up on a range of time scales that starts, as for SOPBNs, at a few hundred milliseconds. It is also interesting that the independent adjustment of synapses connecting inhibitory to layer 2/3 pyramidal neurons in the mouse primary visual cortex has been found to be key for E-I balance to occur on a single-neuron level^51^. These findings concur with the results for the single neuron cross correlation presented in Fig. 7, for which the network average has been performed only as a second step. Furthermore we note that both the self organized bursting states observed in SOPBNs, see Fig. 6, and the alternating up and down states observed for *in vitro* prefrontal and occipital ferret slices are characterized by the asynchronous participation of all neurons^2^.

### Outlook

Which configuration of synaptic weights results from continuously ongoing internal Hebbian learning? We presented here a first inroad into this subject, focusing in particular on the self-organized emergence of E-I balance in terms of large but nearly canceling excitatory and inhibitory inputs. We find that not all self-limiting Hebbian plasticity rules are able to do the job. There is on the other hand no need for a Hebbian learning rule to enforce E-I balance explicitly. We find that E-I balance already emerges when the Hebbian learning rule favors membrane potentials which are small with respect to the variance of the inputs, being nevertheless large enough to be relevant for the neural transfer function.

## Electronic supplementary material

Supplementary Material
